# Characterisation of the SUMO-Like Domains of *Schizosaccharomyces pombe* Rad60

**DOI:** 10.1371/journal.pone.0013009

**Published:** 2010-09-27

**Authors:** Lara K. Boyd, Brenda Mercer, Darren Thompson, Ewan Main, Felicity Z. Watts

**Affiliations:** 1 Genome Damage and Stability Centre, School of Life Sciences, University of Sussex, Brighton, United Kingdom; 2 Division of Biochemistry and Biomedical Sciences, School of Life Sciences, University of Sussex, Brighton, United Kingdom; 3 Division of Chemistry, School of Life Sciences, University of Sussex, Brighton, United Kingdom; University of Minnesota, United States of America

## Abstract

The *S. pombe* Rad60 protein is required for the repair of DNA double strand breaks, recovery from replication arrest, and is essential for cell viability. It has two SUMO-like domains (SLDs) at its C-terminus, an SXS motif and three sequences that have been proposed to be SUMO-binding motifs (SBMs). SMB1 is located in the middle of the protein, SBM2 is in SLD1 and SBM3 is at the C-terminus of SLD2. We have probed the functions of the two SUMO-like domains, SLD1 and SLD2, and the putative SBMs. SLD1 is essential for viability, while SLD2 is not. *rad60-SLD2Δ* cells are sensitive to DNA damaging agents and hydroxyurea. Neither ubiquitin nor SUMO can replace SLD1 or SLD2. Cells in which either SBM1 or SBM2 has been mutated are viable and are wild type for response to MMS and HU. In contrast mutation of SBM3 results in significant sensitivity to MMS and HU. These results indicate that the lethality resulting from deletion of SLD1 is not due to loss of SBM2, but that mutation of SBM3 produces a more severe phenotype than does deletion of SLD2. Using chemical denaturation studies, FPLC and dynamic light scattering we show this is likely due to the destabilisation of SLD2. Thus we propose that the region corresponding to the putative SBM3 forms part of the hydrophobic core of SLD2 and is not a SUMO-interacting motif. Over-expression of Hus5, which is the SUMO conjugating enzyme and known to interact with Rad60, does not rescue *rad60-SLD2Δ*, implying that as well as having a role in the sumoylation process as previously described [1], Rad60 has a Hus5-independent function.

## Introduction

SUMO is a small ubiquitin-like modifier. It is implicated in numerous cellular processes, including chromosome segregation, DNA repair and recombination, and transcriptional control e.g. [2], [3], [4], [5], [6]. More specifically, SUMO-modification of proteins affects protein-protein or protein-DNA interactions e.g. between PCNA and Srs2 in *Saccharomyces cerevisiae* [7], [8] or between thymine DNA glycosylase [9] or mammalian transcription factors, such as p53, Sp3 and Elk-1 and DNA (reviewed in [6], [10]). In addition, it has recently been demonstrated that SUMO-modified proteins interact with SUMO-targeted ubiquitin ligases (STUbLs) that target the modified proteins for proteasomal degradation [11], [12], [13].

SUMO is produced as a precursor protein and processed to the mature form to reveal a diglycine (GG) motif at the C-terminus which is used for attachment to one or more lysine residues in target proteins (reviewed in [10]). Sumoylation requires activation of the mature form of SUMO by a heterodimeric activating (E1) protein. SUMO is then passed to a SUMO conjugating (E2) protein, called Ubc9 or Hus5 in *S. pombe* [14], [15]. SUMO is subsequently attached to target proteins either in a ligase-dependent or -independent manner. In *S. pombe* the SUMO ligases (E3s) are Nse2 and Pli1 [16], [17].

SUMO is capable of forming both covalent and non-covalent interactions with proteins. In many instances, formation of a covalent bond occurs via the lysine residue within the ψKxE consensus motif e.g. [18], [19]. Non-covalent interactions occur via SUMO-interacting motifs (SIMs). The SXS motif is one of two types of SIMs, and was first identified in a peptide derived from the SUMO ligase PIASx in complex with human SUMO-1 [20]. The second type of SIM comprises [V/I]-X-[V/I]-[V/I], and is present in another SUMO ligase, RanBP2, and a variety of proteins including TTRAP and MCAF [21].

Rad60 is a founder member of the RENi (Rad60 Esc2 NIP45) family of proteins which have two SUMO-like domains (SLDs) at the C-terminus [22]. As the name suggests, other members of the RENi family include *S. cerevisiae* Esc2 and human NIP45 [22]. The *ESC2* gene was initially identified in a screen for proteins that restored silencing when tethered to a telomere [23] and more recently has been shown to have a role in genome integrity [24] and S phase repair [25], [26]. NIP45 is implicated as having a function in gene regulation [27]. *S. pombe rad60* is required for response to DNA damaging agents and recovery from S phase arrest [28], [29], [30]. Unlike *S. cerevisiae ESC2*, *rad60* is essential for viability [28].

In addition to the SLDs, Rad60 contains an SXS motif that is thought to be a SIM [31]. It also has three hydrophobic regions that each contain a sequence conforming to the [V/I]-X-[V/I]-[V/I] SIM consensus and these have been termed putative SUMO-binding motifs (SBMs) [31].

Rad60 was originally identified in a screen for mutants defective in homologous recombination (24). It has been proposed that control of Rad60 regulates recombination events when replication is stalled. It is delocalised from the nucleus in an HU-dependent manner on activation of Cds1, the fission yeast S phase checkpoint kinase, but becomes essential for viability on recovery from replication arrest [29]. Genetic and biochemical studies indicate that Rad60 functions with the Smc5/6 (structural maintenance of chromosomes) complex required for recombinational repair and recovery from replication fork stalling [29], [32].

The *S. pombe* Smc5/6 complex comprises eight tightly associated proteins: two large proteins, Smc5 and Smc6, and six smaller, non-SMC proteins, Nse1-6 [33], [34]. All of these proteins apart from Nse5 and Nse6, are essential for viability in *S. pombe*. The role of these proteins is beginning to be elucidated. Nse1 has a RING-like domain frequently associated with ubiquitin E3 ligase activity (e.g. [35]) although no ligase activity has yet been demonstrated for the protein. Nse2 is a SUMO ligase [16], [36], [37]. Nse4 is a kleisin that bridges the Smc5/6 heads [38]. Nse5 and Nse6 form a heterodimer that interacts with the hinge regions of Smc5 and Smc6 [39]. In response to DNA damage, components of the Smc5/6 complex are modified post-translationally by SUMO (e.g. [16], [36], [37]).

In order to further our understanding of the organisation and function of the Smc5/6 complex, we have undertaken a study into the function of domains and motifs in the Rad60 protein. These studies extend those of Raffa et al [31] and Prudden et al [1]. In particular we have investigated the function(s) of the SUMO-like domains (SLDs) and the three putative SUMO binding motifs (SBMs). We show that SLD1 but not SLD2 is essential for viability. Deletion of SLD2 results in sensitivity to DNA damage. We show that while the SLDs resemble SUMO, their function cannot be replaced by SUMO. Additionally, we have analysed the role of three hydrophobic regions that have been proposed to be SBMs. Genetic and biophysical studies indicate that SBM3 is not likely to be a SUMO-interacting motif, but is part of the hydrophobic core of SLD2.

## Materials and Methods

### Strains and plasmids

The strains used in this work are detailed in Table 1. *rad60-SLD2Δ* (sp.1174) and *rad60-FL* (sp.1175) (created as a wild type control for *rad60-SLD2Δ*) were created by the method of Bahler *et al* [40]. The recombinase-mediated cassette exchange (RMCE) system described by Watson et al [41] was used for the creation of other strains. Briefly, a *rad60* haploid ‘base strain’ was created as follows: the *loxP* site was integrated 300bp upstream of the *rad60* coding sequence, and *ura4* ^+^ and the *loxM3* site were integrated immediately downstream of the *rad60* coding sequence. The base strain was checked to ensure that the *rad60* gene was still functional, and that the integration events had not disrupted the function of adjacent genes. A diploid strain heterozygous for this altered *rad60* locus was created by crossing the haploid *h* ^−^ base strain containing the *ade6-210* allele with a *rad* ^+^, *h* ^+^, *ura4-D18*, *leu1-32*, *ade6-216* strain. The base strain (either haploid or diploid as required) was then transformed with wild type and mutant versions of *rad60* flanked by *loxP* and *loxM3* loci, cloned into the *LEU2*-containing plasmid pAW8, and LEU^+^ colonies selected. Recombination was subsequently induced by expression of the Cre recombinase following growth of cells in thiamine-free medium. Strains in which the original copy of *rad60* had been replaced were selected on medium containing 5-FOA. Other plasmids used for *S. pombe* transformation were based on pREP41 or pREP42 [42]. pREP41-*rad60-SLD1Δ* was created by deleting aa 227–308, pREP41-*rad60-SLD2Δ* lacked aa 334–406 and pREP41-*rad60-SLD2Δ-SUMO* contained the coding sequence for aa 1–109 of *S. pombe* SUMO cloned in-frame with *rad60-SLD2Δ* in pREP41-*rad60-SLD2Δ*. *rad60-SLD2Δ-SUMO-M* was created by Quik-Change site-directed mutagenesis (Stratagene) according to the manufacturers instructions. The *hus5* gene was from A Carr (U. of Sussex) [15].

**Table 1:** Strains used in this study.

Strain	Genotype	Reference:
sp.011	*ade6-704*, *ura4-D18*, *leu1-32*, *h^−^*	[52]
sp.432	*rhp51::ura4*, *ade6-704*, *ura4-D18*, *leu1-32*, *h^+^*	[53]
sp.473	*rqh1::ura4*, *ade6-704*, *ura4-D18*, *leu1-32*, *h^−^*	[55]
sp.480	*brc1::LEU2*, *ade6-704*, *ura4-D18*, *leu1-32*, *h^−^*	This work
sp.714	*pli1::ura4*, *ade6-704*, *ura4-D18*, *leu1-32*, *h^−^*	[2]
sp.1123	*nse2-SA*, *ade6-704*, *ura4-D18*, *leu1-32*, *h^−^*	[16]
sp.1125	*smc6-X*, *ade6-704*, *ura4-D18*, *leu1-32*, *h^+^*	[54]
sp.1126	*smc6-74*, *ade6-704*, *ura4-D18*, *leu1-32*, *h^+^*	[46]
sp.1174	*rad60-SLD2Δ*, *ade6-704*, *ura4-D18*, *leu1-32*, *h^−^*	This work
sp.1175	*rad60-FL:kan*, *ade6-704*, *ura4-D18*, *leu1-32*, *h^−^*	This work
sp.1179	*rad60-1*, *ura4-D18*, *leu1-32*, *h^−^*	[28]
sp.1305	*rad60-SLD2Δ*, *nse2-SA*, *ade6-704*, *ura4-D18*, *leu1-32*, *h^−^*	This work
sp.1408	*rad60-SLD2Δ*, *rhp51::ura4*, *ade6-704*, *ura4-D18*, *leu1-32*, *h^+^*	This work
sp.1701	*rad60 base strain*, *ade6-704*, *leu1-32*, *h^−^*	This work
sp.1704	*rad60-SBM2*, *ade6-704*, *ura4-D18*, *leu1-32*, *h^−^*	This work
sp.1778	*rad60-SBM1*, *ade6-704*, *ura4-D18*, *leu1-32*, *h^−^*	This work
sp.1845	*rad60 base strain heterozygous diploid*, *ade6-210*, *ade6-216*, *leu1-32*, *h^+^/h^−^*	This work
sp.1925	*rad60-SBM3*, *ade6-704*, *ura4-D18*, *leu1-32*, *h^−^*	This work
sp.2026	*rad60-SLD2Δ-SUMO*, *ade6-704*, *ura4-D18*, *leu1-32*, *h^−^*	This work
sp.2027	*rad60-SLD2Δ-SUMO-M*, *ade6-704*, *ura4-D18*, *leu1-32*, *h^−^*	This work
sp.2045	*rad60-SBM1*,*SBM2*, *ade6-704*, *ura4-D18*, *leu1-32*, *h^−^*	This work

### Analysis of DNA damage responses

UV irradiation was carried out on freshly plated cells using a Stratagene Stratalinker. Ionising radiation sensitivity was assayed using a ^137^Cs source at a dose of 10 Gymin^−1^. Sensitivities to hydroxyurea (HU) and methyl methanesulphonate (MMS) were analysed on YE agar (YEA) at the doses stated.

### Microscopy

Methanol-fixed cells were stained with DAPI (1 µg/ml) and viewed using an Applied Precision Deltavision Spectris microscope with deconvolution software.

### Protein purification

His-tagged proteins expressed from pET15b, were purified using Ni^2+^ agarose (Novagen) according to the manufacturer's instructions.

### Equilibrium Denaturation Studies

Preparation of samples: A stock solution of guanidinium HCl (8 M) was diluted to obtain a large range of denaturant concentrations using a Hamilton Microlab dispenser; 100 µl of a stock solution of SLD2 protein (9 µM) containing 450 mM phosphate, 9 mM DTT (pH 7.0) was added to each denaturant sample (800 µl). This gave a final buffer concentration of 50 mM phosphate pH 7.0 and a protein concentration of 1 µM. The protein/denaturant solutions were pre-equilibrated at 25°C for at least three hours (This was sufficient time for every solution to reach equilibrium [data not shown]).

Fluorescence measurements: All measurements were performed in a thermostatted cuvette holder at 25°C using Varian Cary Eclipse Fluorescence Spectrophotometer. The excitation wavelength was 280 nm, band passes were set at 5 nm for excitation and emission and the fluorescence was measured at the λ_max_ for the denatured state of 352 nm.

### Equilibrium data analysis

Two state folding model: The entire fluorescence monitored denaturation of SLD2 was fitted to equation (1) using the non-linear regression analysis program *Kaleidagraph* (version 4.0 Synergy Software, PCS Inc.):
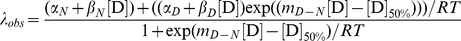
(1)where 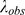 is the observed fluorescence signal, 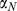 and 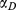 are the intercepts, and 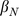 and 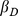 are the slopes of the baselines at the low (N) and high (D) denaturant concentrations, [D]_50%_ is the midpoint of unfolding, [D] is the concentration of denaturant and 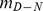 is a constant that is proportional to the increase in degree of exposure of the protein on denaturation.

### Size exclusion chromatography

250 µl of protein was loaded onto a superose 6 column (volume 24 ml) connected to an Amersham Biosciences FPLC and eluted with 20 mM Tris HCl pH 7.9, 150 mM NaCl, 1 mM DTT. Protein elutions were monitored with an in-line UV detector and fractions collected.

### Dynamic Light Scattering

50 µl samples were analyzed at 4°C using a Malvern Instruments Nano S Dynamic Light Scattering instrument. Samples were spun at 14k rpm for 10 minutes and allowed to equilibrate at collection temperature for 2 minutes prior to data collection. Scattering data were analysed for peak position and width to identify particle size and polydispersity.

## Results

### Relationship of the Rad60 SLDs to ubiquitin and SUMO

**Figure 1:**
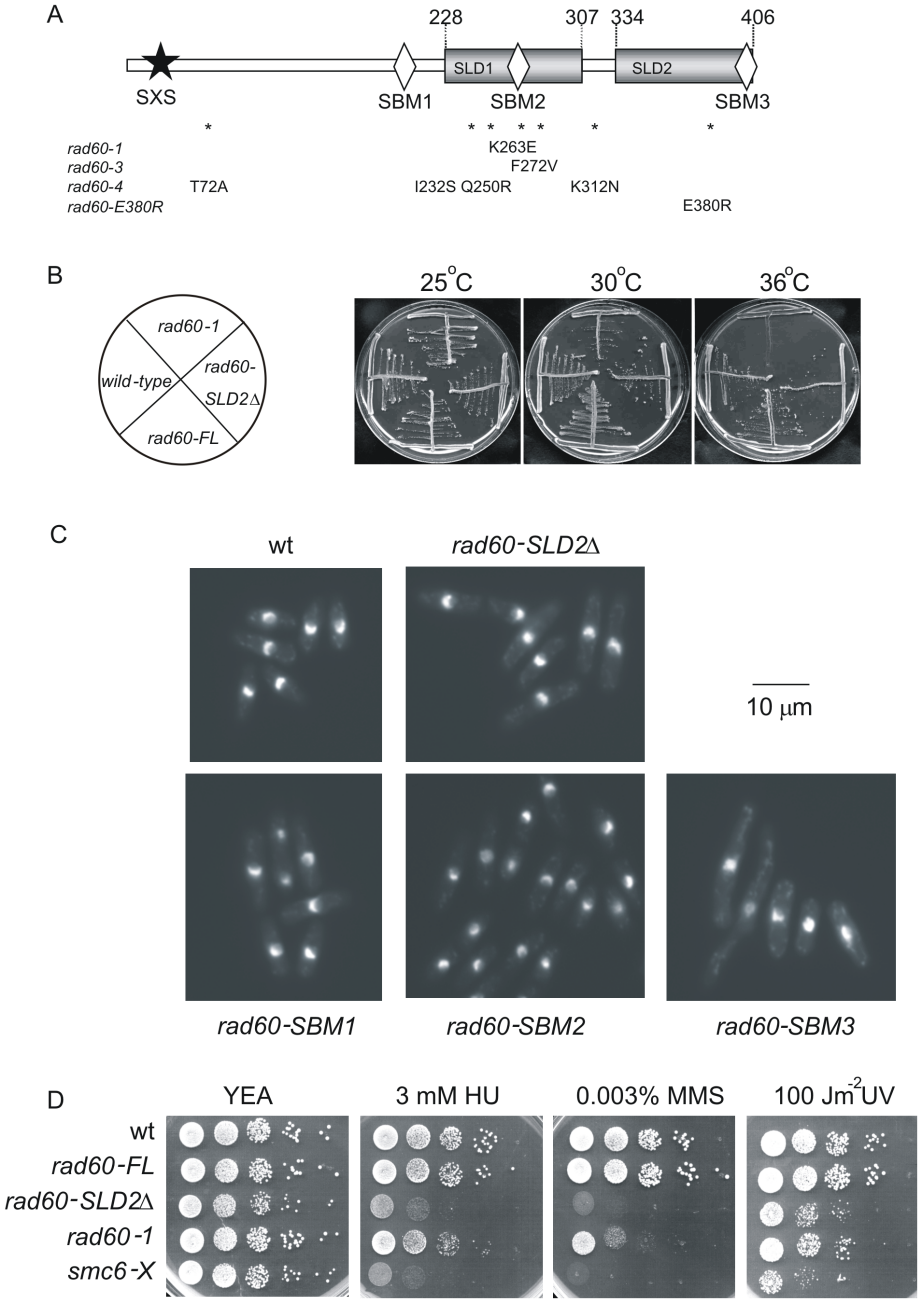
*rad60-SLD2Δ* is *ts* and sensitive to DNA damaging agents. A. Organisation of the Rad60 protein, indicating the position of the SXS motif (star), the putative SBMs (diamonds) and the *rad60* mutations (*). B. *rad60-SLD2Δ* is slightly temperature-sensitive for growth at 36°C. Strains were streaked onto YEA and incubated at the indicated temperatures for 5 days. C. Morphology of DAPI-stained cells. D. Spot tests to assess sensitivity to HU, MMS and UV. 10 µl of serially diluted cells were spotted onto media as indicated. Plates were incubated at 25°C.

Rad60 has two domains (SLD1 and SLD2) at its C-terminus (Figure 1A) that were initially reported to be ubiquitin-like [28]. However, sequence comparisons indicate that SLD2 at least, resembles SUMO more closely than ubiquitin. SLD1 has identity with *S. pombe* ubiquitin and SUMO of 18.4% and 19.7% respectively. For SLD2 the identity with ubiquitin and SUMO is 14.3% and 23.4% respectively. The similarity between SLD2 and SUMO is further demonstrated by the recent publication of the structure of *S. pombe* and human SLD2 [1], [43]. Comparison of the structures of SUMO, ubiquitin and SLD2 and the predicted structure of SLD1 indicates similar overall structures (Figure S1A,B). Interestingly, the amino acids in SLD1 and SLD2 that are the same as, or similar to, amino acids in SUMO, are, in most cases, not the same in the two domains (Figure S2).

### SLD1, but not SLD2 is required for the essential function of Rad60

The importance of the SLDs for Rad60 function is attested to by the fact that the majority of the mutations within three characterised *rad60-ts* mutants lie within SLD1, namely K263E (*rad60-1*) [28], F272V (*rad60-3*), and I232S and Q250R (*rad60-4*) [29] (Figure 1A) (*rad60-4* also contains two mutations outside of SLD1, T72A, and K312N) [29] (Figure 1A). This suggests that SLD1 at least, has a key role in Rad60 function. Additionally, a point mutation within SLD2 (*rad60-E380R*) [1] results in sensitivity to DNA damaging agents. In order to investigate the roles of the SLDs, we attempted to create strains containing versions of Rad60 deleted for both SLD1 and SLD2 and, separately, deleted for a single domain (either SLD1 or SLD2). Using both haploid and diploid strains (see Materials and Methods) we were unable to produce haploid strains in which Rad60 was missing either SLD1+SLD2 (aa 228–406) or missing solely SLD1 (aa 228–307). In contrast, deletion of SLD2 (aa 334–406) resulted in viable cells (*rad60-SLD2Δ*). Thus, consistent with the presence of the *ts* mutations in SLD1, SLD1, but not SLD2, is essential.

### SLD2 is required for response to DNA damaging agents

*rad60-SLD2Δ* is slightly temperature sensitive for growth at 36°C (Figure 1B) when compared to wild-type and *rad60-FL* strains (*rad60-FL* was created in parallel with *rad60-SLD2Δ* as a full length Rad60 control), but less sensitive than *rad60-1*. At permissive temperatures, *rad60-SLD2Δ* cells are slightly elongated compared to wild-type (Figure 1C). *rad60-SLD2Δ* is slightly sensitive to UV (Figure 1D, Figure S3B) and ionising radiation (Figure S3B). However, it is significantly sensitive to HU, (DNA synthesis inhibitor) and MMS (alkylating agent) (Figure 1D) similar to *smc6-X*, which contains a point mutation (R706C) in the hinge region of Smc6 [44], but more sensitive than *rad60-1*. This is consistent with Rad60's reported role in recovery from HU arrest, i.e. in processing of intermediates following exposure to DNA damaging agents or replication fork arrest by HU [30].

To determine whether *rad60-SLD2Δ* behaves differently to other *rad60* mutants we undertook epistasis analysis with *rad60-SLD2Δ* and mutants defective in the Smc5/6 complex and homologous recombination. The results are summarised in Table S1. Consistent with the published analyses of other *rad60* mutants [1], [28], [29], [45], *rad60-SLD2Δ* was synthetically lethal with *smc6-X*, *smc6-74* (contains a point mutation A151T, close to the ATP-binding site [46]), *brc1-d* (deleted for a 6 BRCT domain-containing protein [46]), *rqh1-d* (deleted for the *S. pombe* homologue of the RecQ helicase [47]) and *pli1-d* (deleted for the Pli1 SUMO ligase). Additionally, it is epistatic with *nse2-SA* (contains 2 point mutations, C195S, H197A, in the SP-RING domain of the Nse2 SUMO ligase [16]) and *rhp51-d* (deleted for the Rad51 homologue) (Table S1 and Figure S3). Thus *rad60-SLD2Δ* is a hypomorphic mutant which displays a similar sensitivity to DNA damaging agents or the inhibition of replication and genetic interactions as previously described for *rad60-1*.

### Neither ubiquitin nor SUMO can replace the functions of SLD1 or SLD2

The sequences and structural similarities of the SLDs with ubiquitin and SUMO prompted us to investigate whether the SLDs can be replaced by either ubiquitin or SUMO (both lacking the GG motifs and C-terminal extensions downstream of the GG motifs), or a combination of the two. Figure S4 shows the combinations that we tested. In no case were we able to obtain viable haploid cells with ubiquitin replacing SLD1 or SLD2. Additionally, we were unable to obtain strains in which SLD1 was replaced by SUMO. However, viable cells were obtained when SLD2 was replaced by SUMO (Figure S4, construct 7, *rad60-SLD2Δ-SUMO*).

**Figure 2:**
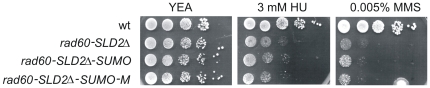
SUMO is unable to functionally replace SLD2. Response of strains, as indicated, to HU and MMS. Plates were incubated at 30°C.

To determine whether SUMO can replace the function of SLD2, *rad60-SLD2Δ-SUMO* was tested for sensitivity to HU and MMS. Figure 2 indicates that *rad60-SLD2Δ-SUMO* has similar sensitivity to HU and MMS as *rad60-SLD2Δ*. To determine why SUMO is not capable of functionally replacing SLD2, differences between the two were sought. While the overall structure of SLD2 resembles that of SUMO, a detailed comparison of the structure of SLD2 with that of SUMO identified some key differences between the two structures [1]. These are (i) that SLD2 lacks the C-terminal tail present in the mature form of SUMO, which is required for interaction with the SUMO activating E1 protein, and (ii) that SUMO has a positively charged cleft formed between β-strand 2 and α-helix 1 which interacts non-covalently with SIMs on interacting proteins. In SLD2 this is obscured by the side chains of P351, F354, R362 and E366. Thus the inability of SUMO to restore wild type function in Rad60-SLD2Δ-SUMO may be due to inappropriate interactions involving SUMO. We therefore introduced a series of mutations into SUMO in *rad60-SLD2Δ-SUMO* to produce *rad60-SLD2Δ-SUMO-M*. The mutant fusion protein lacks two amino acids at the C–terminus of SUMO, namely Q108 and L109 (see Figure S2B) and has four substitutions in amino acids corresponding to those in SLD2 that are proposed to be obscuring the charged cleft, namely K53P, T56F, I64R, R68E. *rad60-SLD2Δ-SUMO-M* was then integrated into the *S. pombe* genome. Figure 2 indicates that the mutations do not restore a wild type response to MMS or HU.

### Intermolecular complementation is not observed with rad60-SLD1 and rad60-SLD2 mutants

**Figure 3:**
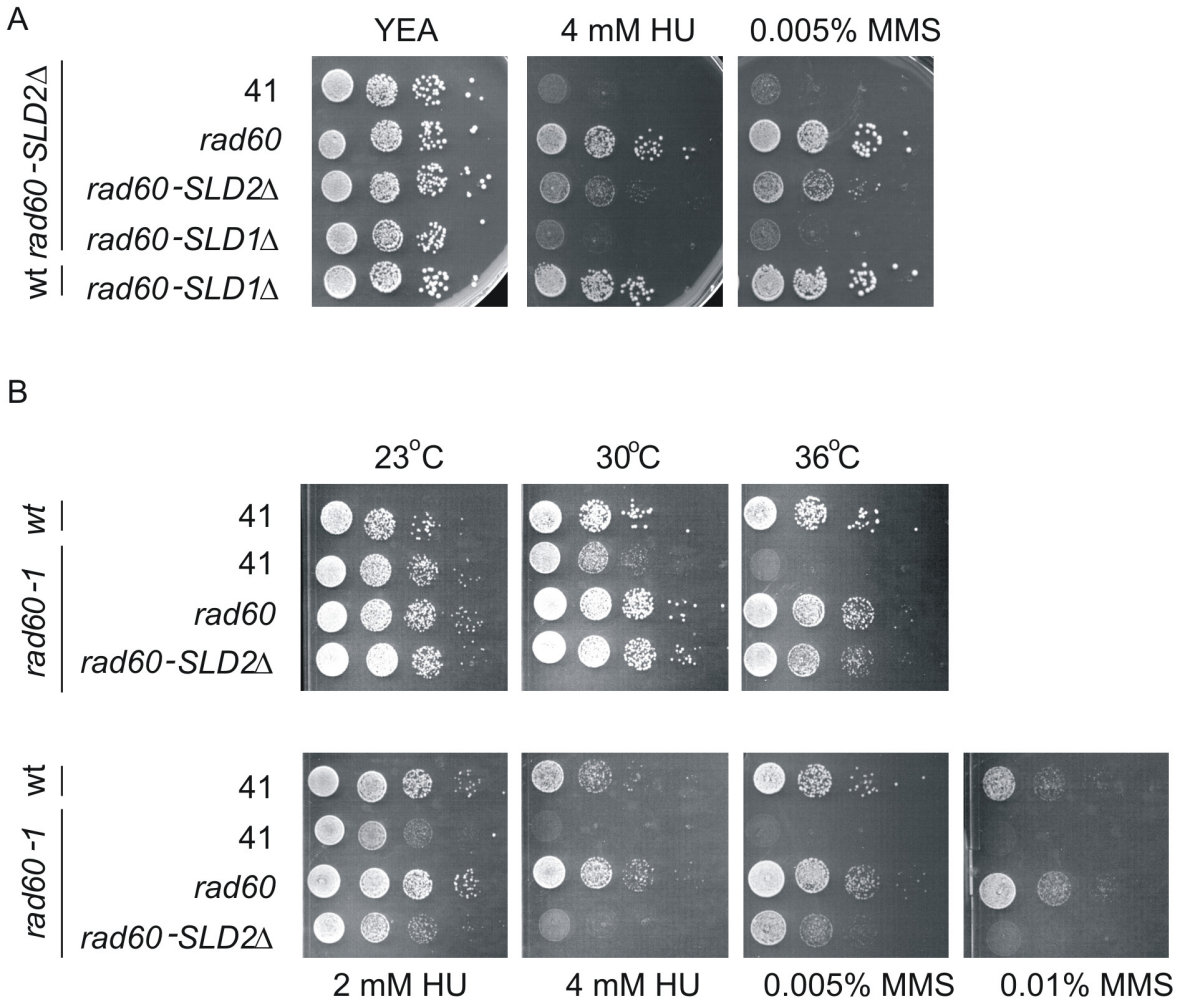
Testing the requirements for Rad60 dimerisation. A. wt and *rad60-SLD2Δ* strains were transformed with pREP41 (41), pREP41-*rad60* (*rad60*), pREP41-*rad60-SLD2Δ* (*rad60-SLD2Δ*) or pREP41-*rad60-SLD1Δ* (*rad60-SLD1Δ*) as indicated. Cells were plated on YEA containing HU and MMS as indicated and incubated at 30°C. B. wt or *rad60-1* cells were transformed with plasmids as indicated. Top row: cells were plated on YEA and incubated at 23°C, 30°C or 36°C as indicated. Bottom row: cells were plated on YEA containing HU or MMS at the doses stated and incubated at 25°C.

Rad60 has been shown to form homodimers via the SLDs [31]. This raises the question as to whether the two molecules both need to contain SLD1 and SLD2. We investigated this by testing whether Rad60 function could be restored through inter-molecular complementation by two Rad60 molecules defective in one case, in SLD1 and in the other in SLD2. Figure 3A indicates that unlike over-expression of full length Rad60, over-expression of Rad60-SLD1Δ (lacking aa 227–308) does not complement the HU and MMS sensitive phenotypes of *rad60-SLD2Δ*.

We extended these studies to test whether Rad60-SLD2Δ can suppress the ts and DNA damage sensitive phenotypes of *rad60-1* (which has a point mutation in SLD1, Figure 1A). As expected, over-expression of full length Rad60 complements the ts and DNA damage sensitivities of *rad60-1* (Figure 3B). In contrast, over-expression of Rad60-SLD2Δ rescues the temperature sensitivity of *rad60-1*, but is less proficient than full length Rad60 in restoring resistance to HU and MMS, particularly at high doses. Since these responses to HU and MMS are similar to those observed when Rad60-SLD2Δ is over-expressed in a *rad60-SLD2Δ* strain (Figure 3A), it is likely that the growth on these plates is due solely to the over-expression of Rad60-SLD2Δ rather than to intramolecular complementation with Rad60-SLD1Δ.

### Probing the role of three putative SUMO binding motifs

It has been proposed that Rad60 contains a SIM (SUMO-interacting motif) in its N-terminus (SXS) [31]. Additionally, three hydrophobic regions within the protein which conform to the [V/I]-X-[V/I]-[V/I] SIM consensus have been identified. These have been termed putative SBMs (SUMO binding motifs), although no interactions with SUMO have been reported for them. Since these putative SBMs are either in, or close to, the SLDs (SBM2 (aa 268–271) lies within SLD1 and SBM3 (aa 401–406) comprises the last six amino acids of SLD2, Figure 1A), we were interested in their contribution to Rad60 function. If these putative SBMs are important motifs it might be expected that they would be highly conserved, at least within *Schizosaccharomyces* species. We therefore compared the sequence of *S. pombe* Rad60 with the recently elucidated Rad60 sequences from *S. japonicus*, *S. cryophilus* and *S. octosporus* (http://www.broadinstitute.org/annotation/genome/schizosaccharomyces_group) (Figure S5). Interestingly, while the SLDs are highly conserved, the regions corresponding to the proposed SBMs are not, particularly SBM1 and SBM2. For example, in *S. cryophilus* and *S. octosporus*, the region corresponding to the putative SBM1 contains Pro, while that corresponding to the putative SBM2 contains Phe. SIMs generally have adjacent acidic sequences e.g. [21]. Only in the case of the putative SBM3 is there a significant stretch of adjacent acidic amino acids, suggesting that SBM1 and 2 may not be SUMO-interacting motifs. Interestingly, the corresponding sequences in *S. cerevisiae* Esc2 are not conserved.

### Rad60 and purified SLD2 do not interact with free SUMO

We next tested whether the putative SBMs interact with SUMO. Using GST-pull down assays (as described in File S1) we do not detect any interaction of full length Rad60 or SLD2 with free SUMO, under conditions where Hus5 and SUMO interact (Figure S6).

### The phenotypes observed for *rad60-SLD1Δ* and *rad60-SLD2Δ* are not due to loss of the SUMO binding motifs SBM2 and SBM3

**Figure 4:**
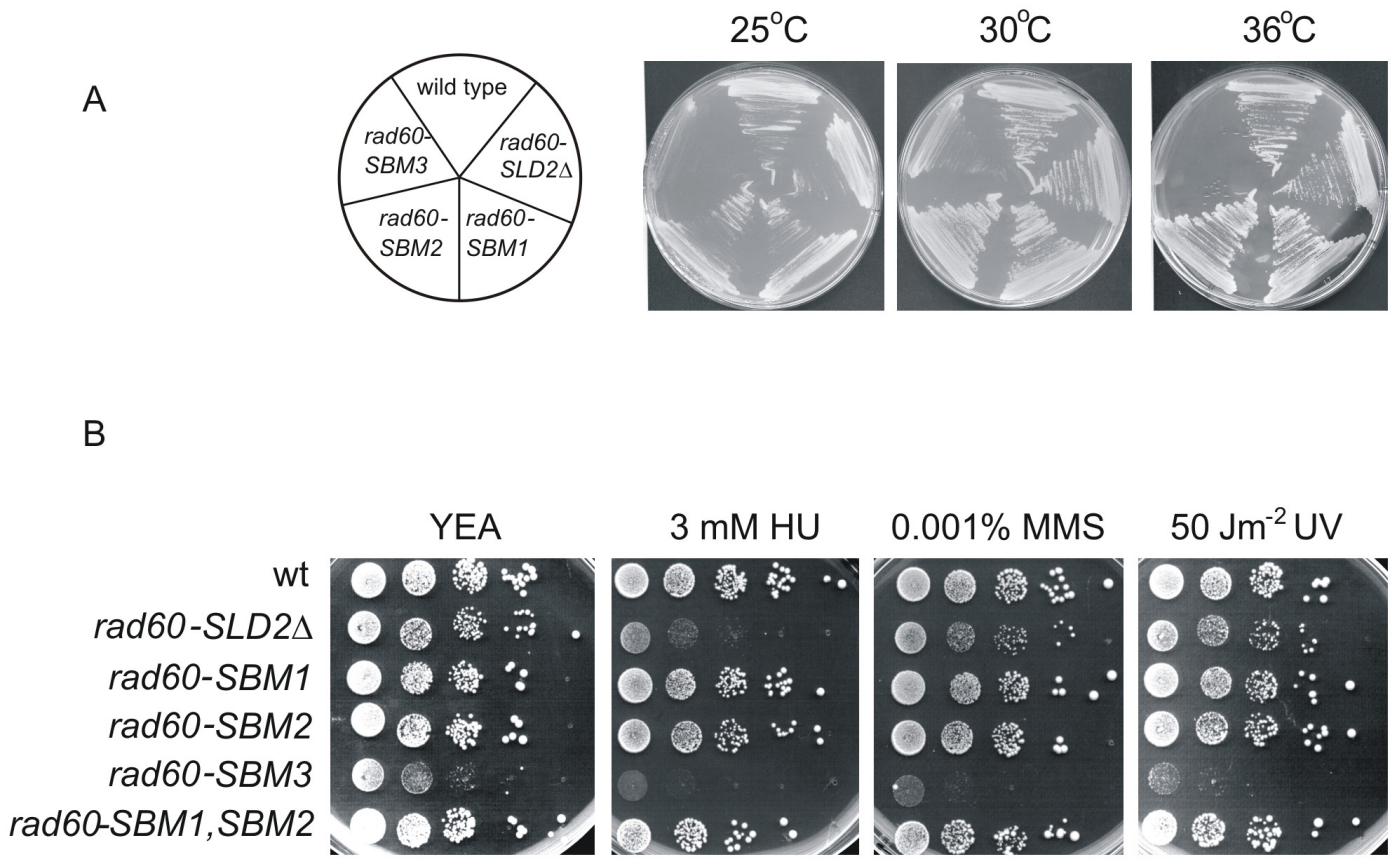
Effect of mutating the three putative Rad60-SBMs. A. *rad60-SBM3* is temperature sensitive. Strains were streaked onto YEA and incubated at the indicated temperatures for 5 days. B. Response of mutants to HU, MMS and UV. 5 fold more cells were plated for *rad60-SBM3* than other strains. Plates were incubated at 30°C for 5 days.

The three putative SBMs are not present in SUMO (Figure S2). Thus, a possible reason for the inability of SUMO to replace the SLDs may be their lack of SBMs. We therefore analysed the effect of mutating SBM2 and SBM3. In addition, we were interested to determine whether the phenotypes that we detect for *rad60-SLD1Δ* and *rad60-SLD2Δ* (namely lethality and sensitivity to DNA damaging agents respectively) are due to deletion of SBM2 or SBM3. We therefore mutated SBM2 (from VVLV to VALA, to produce *rad60-SBM2*), SBM3 (from VSVVLD to ASAVLD, producing *rad60-SBM3*) and in parallel, SBM1 (from ISVV to ISAA, producing *rad60-SBM1*). Mutagenesis of either SBM1 or SBM2 did not have any effect on cell viability, morphology or response to DNA damage (Figure 1C, 4A,B). Mutation of both SBM1 and SBM2 (to produce *rad60-SBM1*,*SBM2*) also had no effect on the response to HU, MMS or UV (Figure 4B). These results indicate that SBM1 and SBM2 do not contribute important functions to the recovery from S phase arrest or the DNA damage response, and do not function redundantly with each other.

In contrast to the results with SBM1 and SBM2 mutants, mutation of SBM3 has a severe effect on cell morphology, growth and response to DNA damaging agents (Figure 1C and 4A,B). *rad60-SBM3* cells are both heat and cold sensitive (25°C and 36°C) (Figure 4A), showing a greater sensitivity to high temperature than *rad60-SLD2Δ*. Thus, mutating SBM3 has a more severe effect on Rad60 function than deletion of the entire SLD2 domain.

**Figure 5:**
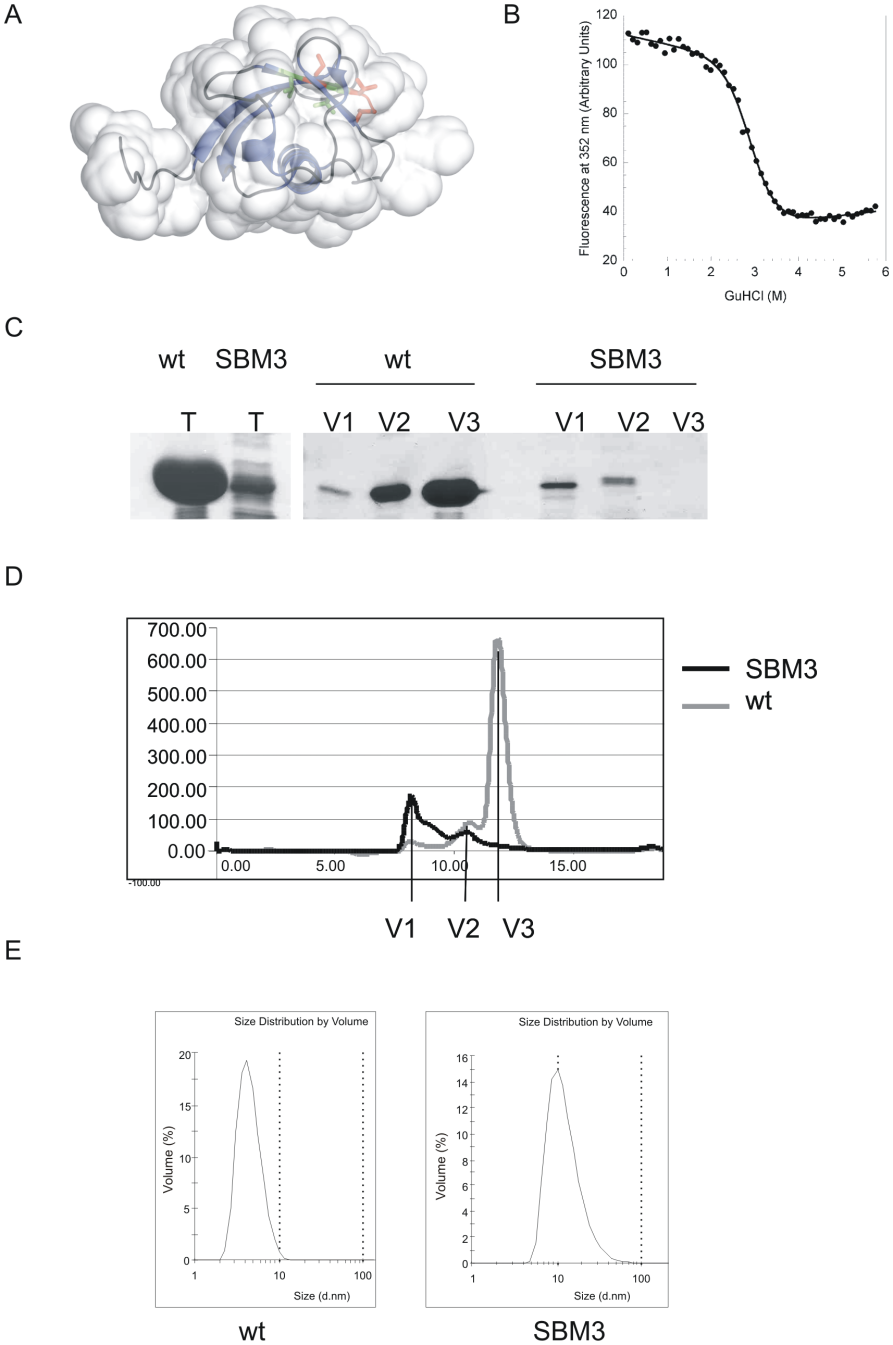
Mutation of SBM3 affects SLD2 structure. A. Position of SBM3 in crystal structure of SLD2 = red and green [1]. SBM3 point mutations created in this study are in green. B. Thermal stability of SLD2. C. SDS PAGE. T = SLD2 protein purified from Ni^+2^ agarose. In both cases (wt and SBM3), 8 µl of 500 µl eluate was loaded onto gel. V1–V3 8 µl of the FPLC fractions indicated in D, was loaded in each case. D. FPLC trace of wt SLD2 and SLD2-SBM3 mutant on Superose 6. SBM3 shows an elution peak after 7 ml whereas the wild type shows elution peaks at 11 ml and 12 ml). E. Dynamic Light Scattering spectra showing solution sizes of wild type and SBM3. The wild type shows a peak indicating a size of diameter 4 nm whereas SBM3 shows a peak indicating a size of diameter of 10 nm.

Since the structure of Rad60 SLD2 has recently been determined [1] we are able to map the positions of the amino acids in SBM3 that we have mutated (Figure 5A). This shows that they are located within the hydrophobic core of the protein and are completely buried. They are both, therefore, likely to be critical for the stability of the domain. To define the effect of these mutations on the Rad60 protein, we determined the stability of SLD2 using a chemical denaturation assay at 298K. We found this to be 6.2 kcalmol^−1^ (Figure 5B). It has been shown that removing individual core residues generally leads to a loss of stability of at least 1 kcalmol^−1^ per methylene group removed [48], [49], [50]. Thus, as the two amino acid substitutions in SBM3 each remove four methylene groups, it is likely that in the *rad60-SMB3* mutant, the SLD2 domain would be completely unfolded.

To further investigate the effect of the SBM3 mutation on the stability of SLD2, we attempted to purify SLD2-SBM3. Using our standard conditions for over-expression in *E. coli*, where the majority of wild type SLD2 is soluble, we observed that SLD2-SBM3 is predominantly in the insoluble fraction (data not shown). However, a small amount of soluble mutant protein was purified (Figure 5C). Analysis of wild type and SBM3 mutant forms of SLD2 were then analysed by size exclusion chromatography (Figure 5D). The majority of wild type SLD2 migrated as a discrete peak (V3), while most of the SBM3 mutant form of SLD2 eluted in the void volume (V1). SDS PAGE (Figure 5C) confirms that the majority of wild type SLD2 is in V3, and that the SBM3 mutant form is mainly present in high Mr fractions (V1–V2), but not in V3 as is the case with the wild type SLD2. This suggests that the SBM3 mutant form of SLD2 forms soluble aggregates. This was confirmed using dynamic light scattering (Figure 5E). The two samples clearly show peaks at different positions, the wild type giving a calculated diameter of 4 nm and the mutant a diameter of 10 nm. This suggests an increase in volume of 16 times. Thus the severe phenotype that we observe for *rad60-SBM3* is likely due to misfolding of SLD2.

### Genetic relationship of *rad60-SLD2Δ* with components of the sumoylation system

**Figure 6:**
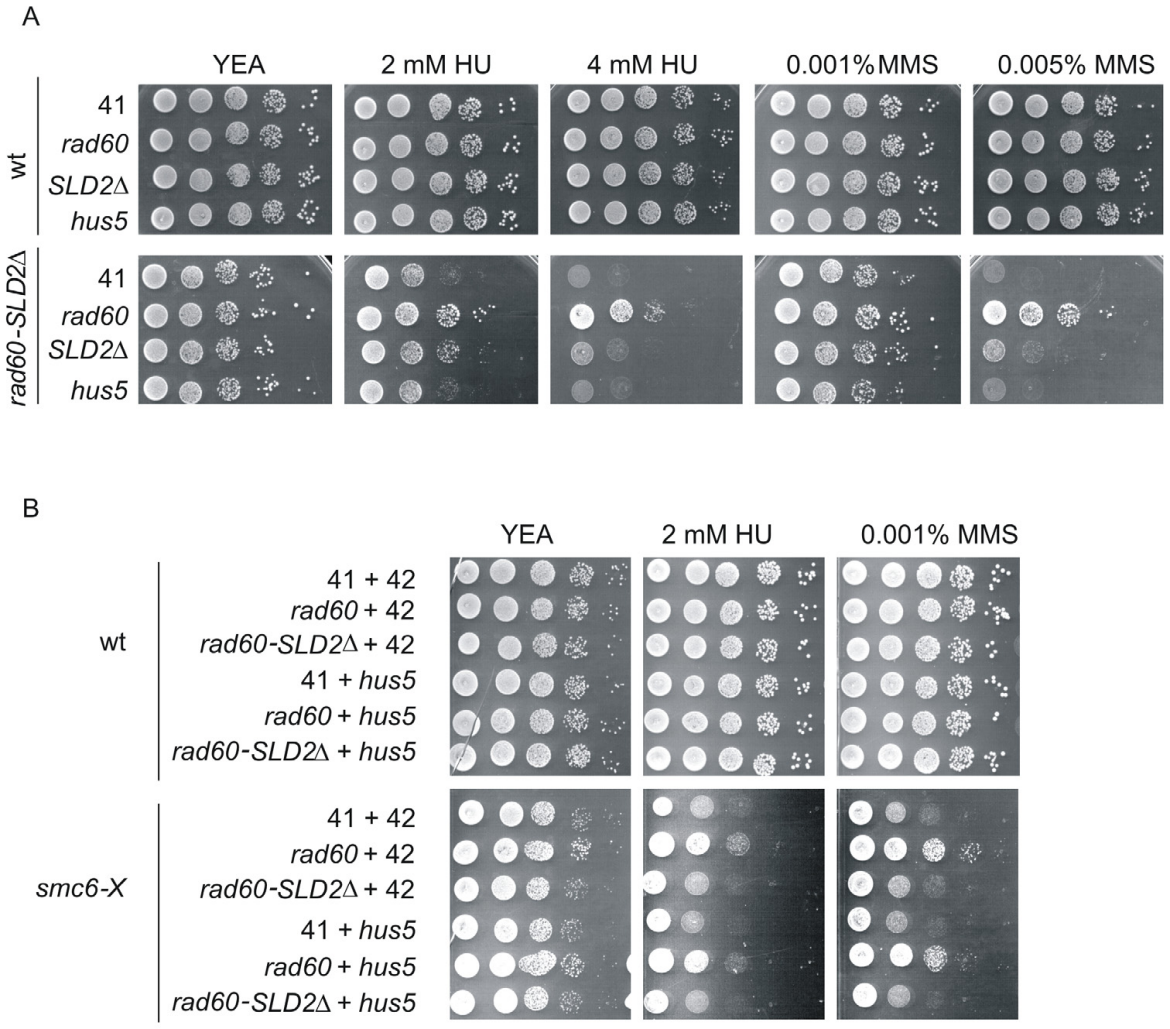
Relationship of *Rad60-SLD2Δ* to Hus5. A. wt and *rad60-SLD2Δ* cells were transformed with pREP41 (41), pREP41-*rad60* (*rad60*) or pREP41-*rad60-SLD2Δ* (*SLD2Δ*) or pREP41-Hus5 (*hus5*) as indicated. Cells were plated on YEA with supplements at 30°C. B. wt and *smc6-X* cells were transformed with combinations of pREP41 (41), pREP42 (42), pREP41-*rad60* (*rad60*), pREP41-*rad60-SLD2Δ* (*SLD2Δ*), pREP42-*hus5* (*hus5*) as indicated.

The Rad60 SLDs interact with components of the sumoylation machinery [1]. In particular, the SLD2s of Rad60, Esc2 and Nip45 interact with the SUMO conjugating enzyme (E2), Hus5/Ubc9 [1], [25], [26], [43]. The *hus5-62* strain is extremely slow growing and prone to accumulate suppressors, making it unreliable to use for epistasis analysis. To overcome these problems, we investigated whether over-expressing Hus5 in *rad60-SLD2Δ* could rescue the sensitivities to HU and MMS. Wild type and *rad60-SLD2Δ* cells were transformed with pREP41-Hus5 and the effect compared with over-expression of full-length Rad60 and Rad60-SLD2Δ. Wild type cells were not affected by over-expression of any versions of Rad60 or Hus5 (Figure 6A,B upper panel). As expected, over-expression of full length Rad60 reverses the HU and MMS sensitivities of *rad60-SLD2Δ* cells. However, over-expression of Hus5 does not reverse this phenotype. This supports the hypothesis that while SLD2 and Hus5 interact, SLD2 has some functions independent of the sumoylation system.

Over-expression of full length Rad60 has previously been shown to partially rescue the MMS sensitivity of *smc6-X* [28]. We next investigated whether over-expression of Rad60-SLD2Δ has any effect in *smc6-X*. We confirm that over-expression of Rad60 can reverse the sensitivity of *smc6-X* to MMS, and has a slight effect on the response to HU (Figure 6B). In contrast, over-expression of Rad60-SLD2Δ is unable to rescue these sensitivities. We next tested the effect of over-expression of Hus5. Over-expression of Hus5 either on its own, or with Rad60, has no effect on the response of *smc6-X* to HU or MMS. Additionally, over-expression of Hus5 with Rad60-SLD2Δ does not restore resistance to HU or MMS in *smc6-X*. This is consistent with the proposal that Rad60 has function(s) independent of the sumoylation system.

## Discussion

In this study we have analysed the requirement for the SUMO-like domains (SLDs) and the putative SBMs for Rad60 function. We show that SLD1 is essential for cell viability under normal growth conditions, whereas SLD2 is not. Deletion of SLD2 results in slight temperature sensitivity and sensitivity to DNA damaging agents, particularly MMS, and the DNA synthesis inhibitor, HU.

We show that despite the structural similarities with ubiquitin and SUMO, the functions of SLD1 and SLD2 cannot be provided by either ubiquitin or SUMO. Since the Rad60 SLDs interact with components of the SUMO modification machinery [1], it is perhaps not surprising that ubiquitin cannot substitute for either of the SLDs. In contrast, since the SLDs more closely resemble SUMO, the reason for the inability of SUMO to functionally replace either or both SLDs in Rad60 is less clear, particularly since a single copy of SUMO can functionally replace the two SLDs in *S. cerevisiae* Esc2 [24].

We tested whether the inability of SUMO to replace SLD2 is due to inappropriate interactions involving SUMO, by removing two amino acids (Q108, L109) from the C-terminus that are required for interaction of SUMO with the E1, and then mutated four amino acids in the region required for interaction with SUMO-interacting motifs (SIMs). Mutation of these regions in SUMO in *rad60-SLD2Δ-SUMO-M* did not restore wild type function to the hybrid molecule and thus imply a specific role for SLD2 not undertaken by SUMO.

Two possible explanations for the ability of SUMO to replace SLD2 in Esc2 but not in Rad60 are that either, the similarity between *S. cerevisiae* SUMO and Esc2 SLD2 is greater than that between *S. pombe* SUMO and Rad60 SLD2, or that Esc2 and Rad60 have somewhat different roles in cells, such that SUMO can replace the SLDs in Esc2, but not in Rad60. Pair wise sequence comparisons do not indicate gross differences in similarities between the SLDs and the respective SUMO sequences (Esc2 SLD2 and SUMO are 17.6% identical and 40% similar, while Rad60 SLD2 and SUMO are 20% identical and 36.9% similar). This suggests that sequence similarity may not account for the ability of SUMO to replace the Esc2 SLDs, although it is possible that certain key epitopes in Esc2 SLD2 may be present in *S. cerevisiae* SUMO, while the same may not be true for Rad60 SLD2 and *S. pombe* SUMO. Alternatively, and our preferred hypothesis, the difference may be related to the different functions of Esc2 and Rad60 in cells. Rad60 is essential for cell viability, while Esc2 is not. Additionally, an *esc2* null mutant is sensitive to MMS but not to HU, UV or IR, unlike *rad60* mutants. It has therefore been proposed that Esc2 probably acts to prevent, or process only limited types of DNA damage, unlike the case with Rad60 [26]. This suggests that Rad60 may be involved in a more complex set of molecular interactions than is Esc2. Despite the likely similarity in structure, the two SLDs in Rad60 have been demonstrated to be involved in distinctly different interactions with components of the sumoylation pathway [1]. This may account for the fact that Rad60 needs to contain two SLDs neither of which can be replaced by SUMO,

Raffa et al [31] demonstrated that Rad60 homodimerises via the SLDs. We observe using FPLC and GST-pulldowns (data not shown) that SLD2 does not interact with itself. This suggests that homodimerisation occurs either between two SLD1s or between SLD1 and SLD2. We tested this latter possibility by investigating whether intermolecular complementation occurred between two mutant Rad60 proteins defective in one case in SLD1 and in the other in SLD2. No intermolecular complementation was observed. This suggest two possibilities. The first is that homodimerisation occurs between two SLD1s. As SLD1 protein is not very soluble we have been unable to test this. The second possibility is, that since our assay is for Rad60 function and not specifically for homodimerisation, that a Rad60 function unrelated to homodimerisation, e. g. involving intramolecular folding, requires that both SLD1 and SLD2 need to be present in the same molecule. This issue will be resolved with the elucidation of the crystal structure of the full-length Rad60 protein.

Raffa et al [31] proposed that three hydrophobic regions in Rad60 were putative SUMO binding motifs (SBMs), and that SBM3 is required for homodimerisation. We have tested the requirement for these putative SBMs *in vivo*. Mutation of SBM1 and SBM2 has no effect on cell viability or DNA damage responses. Since mutation of SBM2 results in viable cells, removal of this SBM likely does not account for the loss of viability observed in *rad60-SLD1Δ* cells, and the inability of SUMO to substitute for SLD1. Since some proteins (e.g. STUbLs) contain more than one SIM (e.g. [51] ) we tested the effect of mutating both SBM1 and SBM2. Since the *rad60-SBM1*,*SBM2* double mutant grows as wild type and is not sensitive to HU or MMS, we conclude that SBM1 and SBM2 do not function redundantly. In contrast to the results with the SBM1 and SBM2 mutants, we see a striking effect when we mutate two residues in SBM3. From the published structure of SLD2 and our results from chemical denaturation studies we propose that the mutations would drastically affect the stability of SLD2, with the likely result that the domain would not be correctly folded. This is also likely to be the case in [31], where 6 aa (comprising an entire β-sheet) were deleted from the C-terminus of Rad60.

Since SLD2 interacts with Ubc9/Hus5 [1], [25], [26], it has been proposed that Rad60 may recruit SUMO-charged Ubc9 to mediate sumoylation of specific proteins, or that it may sequester Ubc9 in an inactive complex to down-regulate sumoylation [1]. We observe that over-expression of Hus5 does not rescue the phenotypes of *rad60-SLD2Δ*. Thus, SLD2 likely has a function, in addition to its role in sumoylation, that is independent of Hus5. This conclusion is supported by the fact that, while over-expression of full length Rad60 suppresses the HU and MMS sensitivity of *smc6-X*, co-over-expression of Hus5 and Rad60-SLD2Δ in *smc6-X* does not. This suggests that Rad60 function is not simply to recruit the SUMO conjugating enzyme Hus5 to the Smc5/6 complex and into close proximity with the Nse2 SUMO ligase subunit. The viability and mild DNA damage sensitivities of the SUMO ligase dead *nse2-SA* mutant [16] is further support for both Smc5/6 and Rad60 having functions independent of the sumoylation system.

In conclusion, we have demonstrated that SLD1 but not SLD2 is required for the essential function of Rad60, and that neither can be replaced by ubiquitin or SUMO. Mutational analysis indicates that the inability of SUMO to functionally replace SLD2 is not due to the slightly extended C–terminus or the presence of the SIM-interacting region. *rad60-SLD2Δ* is sensitive to HU and MMS. Mutation of the SBMs indicates that neither SBM1 nor SMB2 is required for the DNA damage response. Since mutation of SBM3, which is present in the hydrophobic core of SLD2, destabilises SLD2, we conclude that SBM3 does not interact with SUMO, but is required for maintaining SLD2 structure. Our over-expression studies indicate that although SLD2 interacts with the SUMO conjugating enzyme Hus5/Ubc9, Rad60 also has a Hus5/sumoylation-independent role.

## Supporting Information

File S1 Supplementary materials and methods.
(0.03 MB DOC)

Table S1 Epistasis analysis of rad60-SLD2Δ-S. E = epistatic.
(0.03 MB DOC)

Figure S1 Comparison of actual structures of ubiquitin, SUMO, Rad60-SLD2 and the predicted structure of Rad60-SLD1. Human ubiquitin (ubiquitin): 1ubq, human SUMO-1 (SUMO): 2asq, Rad60-SLD2 (SLD2): 3GOE. The models were aligned using the least-squares fit program for the whole polypeptide. A. Position of α-helices and β-sheets. B. Surface charge, red negative, blue positive.
(1.85 MB TIF)

Figure S2 Alignment of Rad60-SLD1 and -SLD2 with SUMO and each other. Hs = human, Sp = *S. pombe*. # = positions of putative SBMs in SLD1 and SLD2. * = amino acids conserved between SLDs but not with SUMO. $ = aa removed in Rad60-SLD2Δ-SUMO-M, ∼ = aa mutated in Rad60-SLD2Δ-SUMO-M.
(0.03 MB DOC)

Figure S3 Response of *rad60-SLD2Δ* to DNA damaging agents. A. and B. Epistasis analysis with *nse2-SA* and *rhp51-d*. A. Spot tests. B. Survival curves. Experiments were done in triplicate. Averages and standard deviations plotted.
(1.51 MB TIF)

Figure S4 Scheme indicating ubiquitin and SUMO replacement constructs. Constructs were created in pAW8 (37) and used to transform haploid and diploid *rad60* base strains. Star = SXS motif, diamond = putative SBM.
(0.47 MB TIF)

Figure S5 Alignment of Rad60 sequences. Scry = *S. cryophilus*, Soct = *S. octosporos*, Spom = *S. pombe*, Sjap = *S. japonicus*, Scer = *S. cerevisiae*. # = putative SBMs.
(0.03 MB DOC)

Figure S6 Rad60 and SLD2 do not interact with free SUMO GST pulldown assays. A. GST-Rad60 + His-SUMO. B. GST-Rad60-SLD2 + His-SUMO, GST-Hus5 + His-SUMO. G = GST, S = SUMO, I = input, U = unbound, B = bound.
(1.09 MB TIF)
