# Supplementary material for: Characterisation of the SUMO-Like Domains of Schizosaccharomyces pombe Rad60
Source: PLoS One. 2010 Sep 27;5(9):e13009. doi: 10.1371/journal.pone.0013009 (PMC2946365; doi:10.1371/journal.pone.0013009)
Supplement: File S1 — Supplementary materials and methods. (0.03 MB DOC) [file pone.0013009.s001.doc]

**Supplementary Information**

**Materials and Methods**

*Molecular modeling*

Molecular modelling of the structures was undertaken using the CCP4MG programme.

*Sequence alignments*

Sequences were aligned using the Clustal W program.

*GST-pulldown assays*

GST-tagged proteins were expressed from pGEXKGH using glutathione Sepharose (Pharmacia) according to the manufacturer’s instructions. GST pull-down assays were conducted as described previously [1]. They involved either the mixing of purified protein with extracts of cells expressing GST or His-tagged protein, or the mixing of purified proteins. GST-tagged proteins were then purified using glutathione Sepharose, followed by SDS–PAGE.

**Figure Legends**

**Supplementary Figure 1**

Comparison of actual structures of ubiquitin, SUMO, Rad60-SLD2 and the predicted structure of Rad60-SLD1

Human ubiquitin; 1ubq, human SUMO-1: 2asq, Rad60-SLD2:3GOE. The models were aligned using the least-squares fit program for the whole polypeptide. A. Position of α-helices and β-sheets. B. Surface charge, red negative, blue positive.

**Supplementary Figure 2**

Alignment of Rad60-SLD1 and –SLD2 with SUMO and each other.

Hs = human, Sp = *S. pombe*. # = positions of putative SBMs in SLD1 and SLD2. * = amino acids conserved between SLDs but not with SUMO. $ = aa removed in *rad60-SLD2Δ-SUMO-M*, ~ = aa mutated in *rad60-SLD2Δ-SUMO-M.*

**Supplementary Figure 3**

Response of *rad60-SLD2Δ* to DNA damaging agents.

A. and B. Epistasis analysis with *nse2-SA and rhp51-d*. A. Spot tests. B. Survival curves. Experiments were done in triplicate. Averages and standard deviations plotted.

**Supplementary Figure 4**

Scheme indicating ubiquitin and SUMO replacement constructs.

Constructs were created in pAW8 (37) and used to transform haploid and diploid *rad60* base strains. Star = SXS motif, diamond = putative SBM.

**Supplementary Figure 5**

Alignment of Rad60 sequences

Scry = *S. cryophilus, Soct = S. octosporos*, Spom = *S. pombe*, Sjap = *S. japonicus*, Scer = *S. cerevisiae*. # = putative SBMs.

**Supplementary Figure 6**

Rad60 and SLD2 do not interact with free SUMO

GST pulldown assays. A. GST-Rad60 + His-SUMO. B. GST-Rad60-SLD2 + His-SUMO, GST-Hus5 + His-SUMO. G = GST, S = SUMO, I = input, U = unbound, B = bound.

**Supplementary Table 1**

Epistasis analysis of *rad60-SLD2Δ*

E = epistatic.

**References**

1. Ho JC, Warr NJ, Shimizu H, Watts FZ (2001) SUMO modification of Rad22, the Schizosaccharomyces pombe homologue of the recombination protein Rad52. Nucleic Acids Res 29: 4179-4186.
