# Supplementary material for: Characterisation of the SUMO-Like Domains of Schizosaccharomyces pombe Rad60
Source: PLoS One. 2010 Sep 27;5(9):e13009. doi: 10.1371/journal.pone.0013009 (PMC2946365; doi:10.1371/journal.pone.0013009)
Supplement: Table S1 — Epistasis analysis of rad60-SLD2Δ-S. E = epistatic. (0.03 MB DOC) [file pone.0013009.s002.doc]

**Table S1**

Summary of epistasis analysis

| **Double with** | **Function** | **viable** | **UV** | **IR** | **HU** | **MMS** |
| --- | --- | --- | --- | --- | --- | --- |
| *smc6-X* | Smc5/6 complex member | No | - | - | - | - |
| *smc6-74* | Smc5/6 complex member | No | - | - | - | - |
| *nse2-SA* | Smc5/6 complex member  SUMO ligase | Yes | E | E | E | E |
| *brc1-d* | BRCT domain protein | No | - | - | - | - |
| *rhp51−d* | Homologous recombination | Yes | E | E | E | E |
| *rqh1-d* | RecQ helicase | No | - | - | - | - |
| *pli1-d* | SUMO ligase | No | - | - | - | - |
