# Supplementary figures and images for: Characterisation of the SUMO-Like Domains of Schizosaccharomyces pombe Rad60
Source: PLoS One. 2010 Sep 27;5(9):e13009. doi: 10.1371/journal.pone.0013009 (PMC2946365; doi:10.1371/journal.pone.0013009)

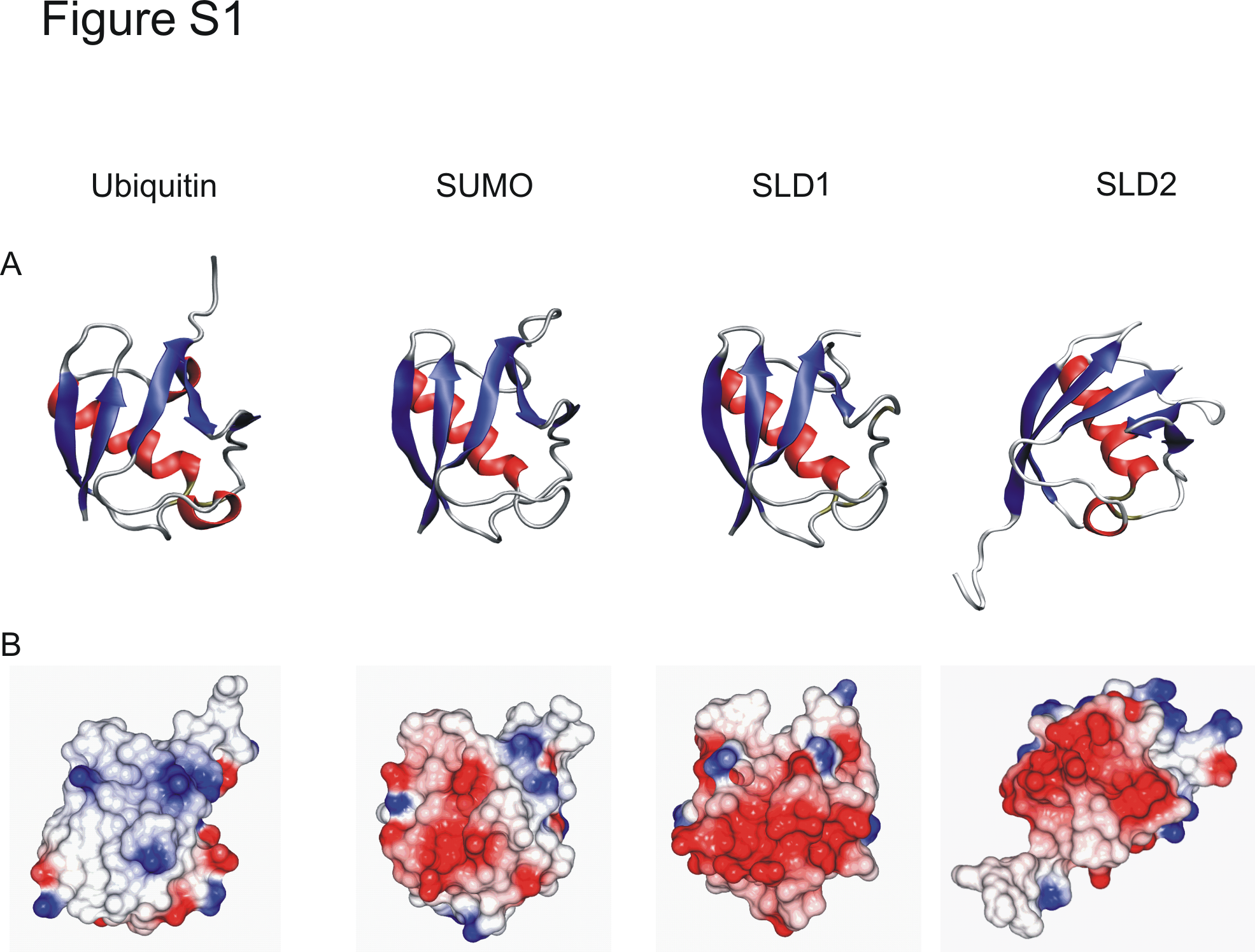

Supplement: Figure S1 — Comparison of actual structures of ubiquitin, SUMO, Rad60-SLD2 and the predicted structure of Rad60-SLD1. Human ubiquitin (ubiquitin): 1ubq, human SUMO-1 (SUMO): 2asq, Rad60-SLD2 (SLD2): 3GOE. The models were aligned using the least-squares fit program for the whole polypeptide. A. Position of α-helices and β-sheets. B. Surface charge, red negative, blue positive. (1.85 MB TIF) [file pone.0013009.s003.tif]

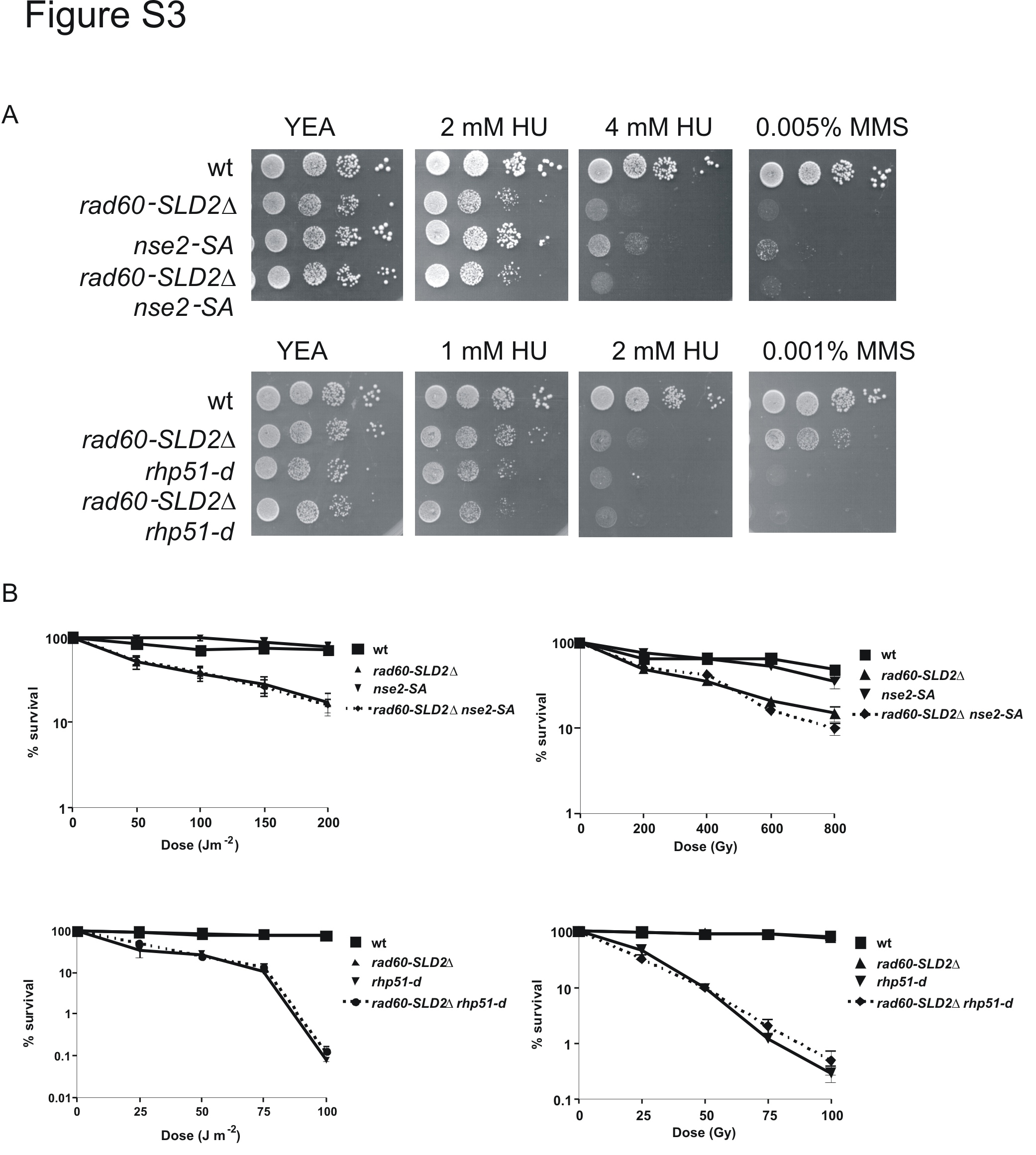

Supplement: Figure S3 — Response of rad60-SLD2Δ to DNA damaging agents. A. and B. Epistasis analysis with nse2-SA and rhp51-d. A. Spot tests. B. Survival curves. Experiments were done in triplicate. Averages and standard deviations plotted. (1.51 MB TIF) [file pone.0013009.s005.tif]

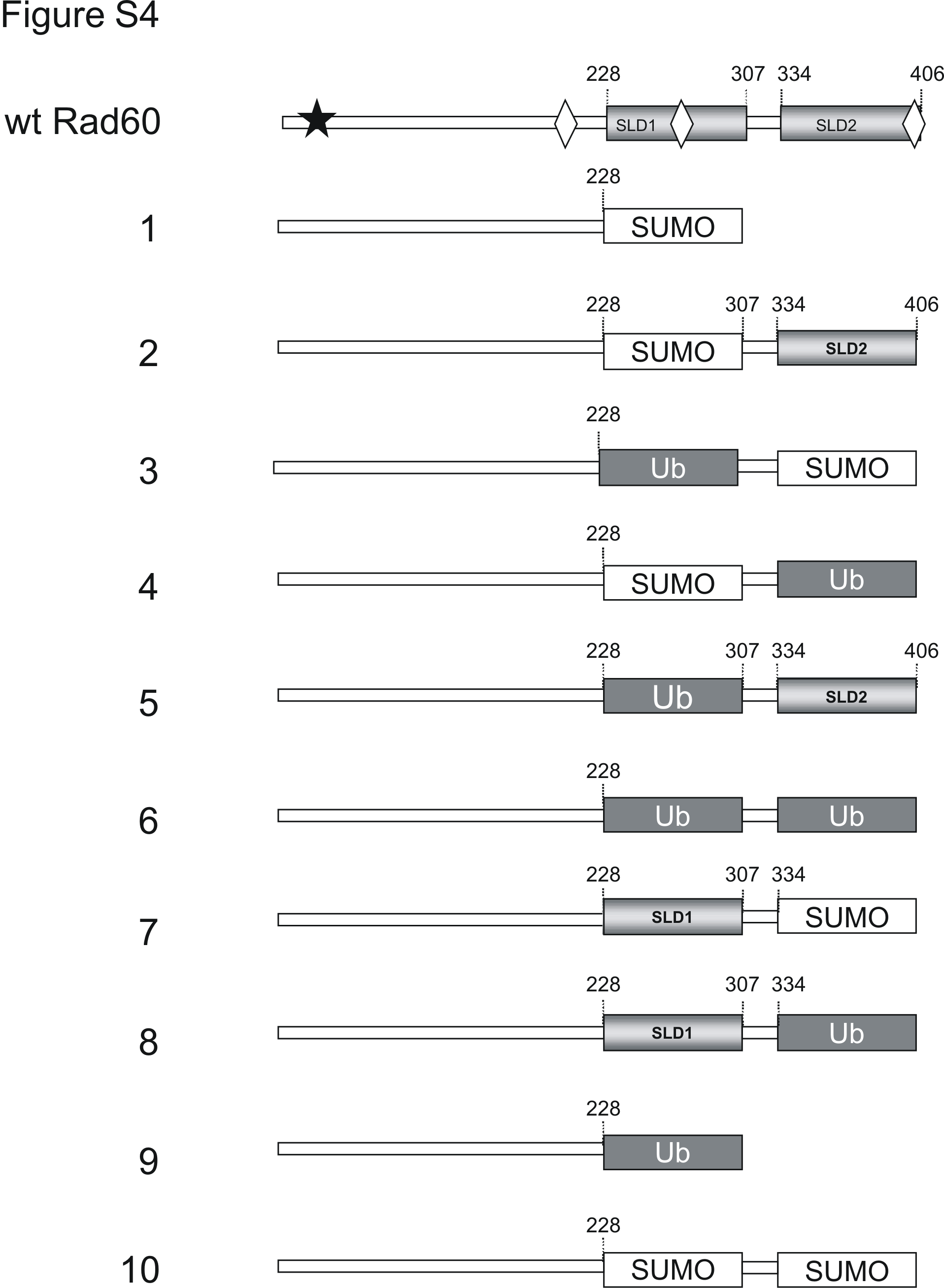

Supplement: Figure S4 — Scheme indicating ubiquitin and SUMO replacement constructs. Constructs were created in pAW8 (37) and used to transform haploid and diploid rad60 base strains. Star = SXS motif, diamond = putative SBM. (0.47 MB TIF) [file pone.0013009.s006.tif]

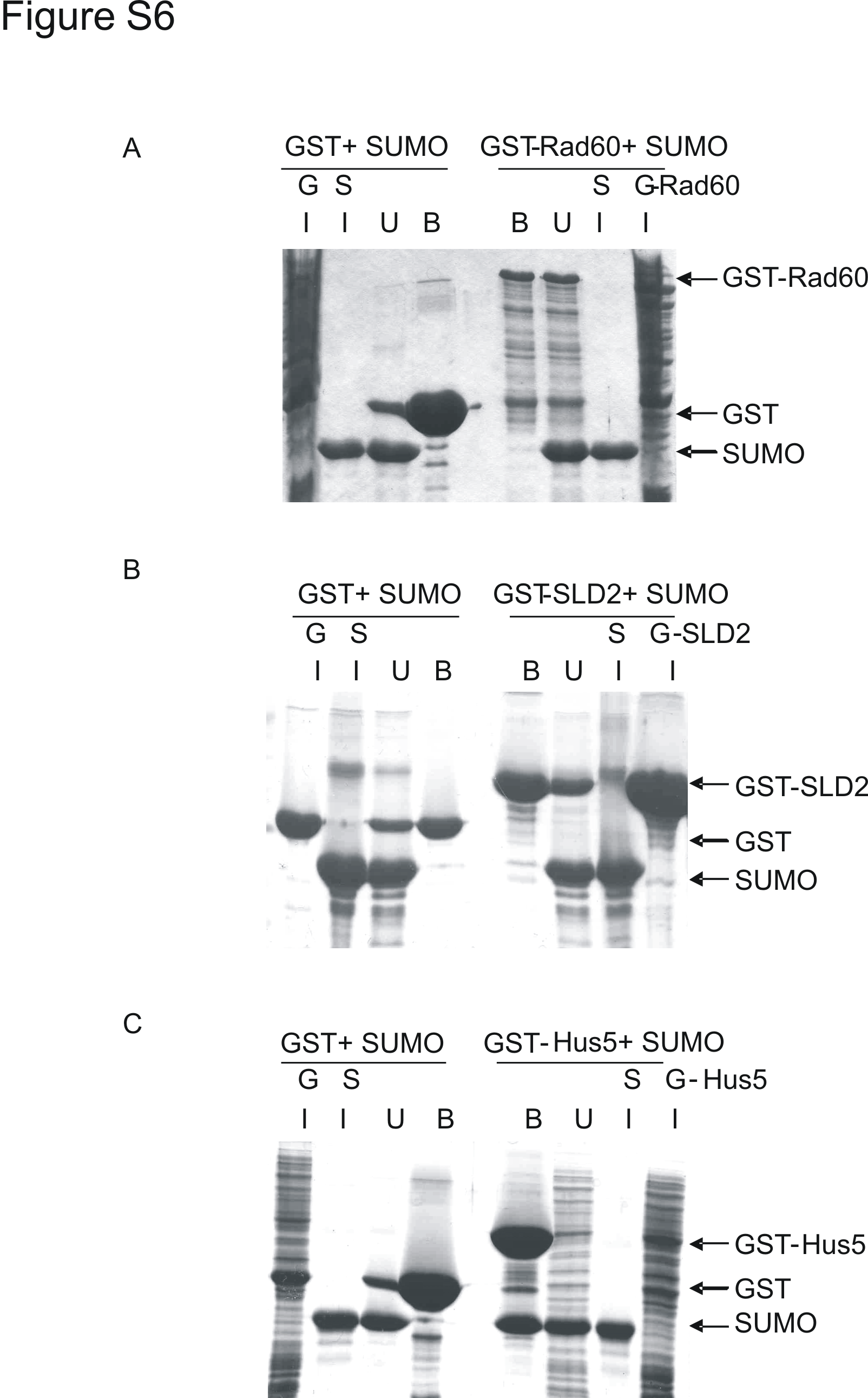

Supplement: Figure S6 — Rad60 and SLD2 do not interact with free SUMO GST pulldown assays. A. GST-Rad60 + His-SUMO. B. GST-Rad60-SLD2 + His-SUMO, GST-Hus5 + His-SUMO. G = GST, S = SUMO, I = input, U = unbound, B = bound. (1.09 MB TIF) [file pone.0013009.s008.tif]
