# Supplementary material for: Characterisation of the SUMO-Like Domains of Schizosaccharomyces pombe Rad60
Source: PLoS One. 2010 Sep 27;5(9):e13009. doi: 10.1371/journal.pone.0013009 (PMC2946365; doi:10.1371/journal.pone.0013009)
Supplement: Figure S2 — Alignment of Rad60-SLD1 and -SLD2 with SUMO and each other. Hs = human, Sp = S. pombe. # = positions of putative SBMs in SLD1 and SLD2. * = amino acids conserved between SLDs but not with SUMO. $ = aa removed in Rad60-SLD2Δ-SUMO-M, ∼ = aa mutated in Rad60-SLD2Δ-SUMO-M. (0.03 MB DOC) [file pone.0013009.s004.doc]

Figure S2

A

Hs_SUMO-1 1 ----------MSDQEAKPSTEDLGD------KKEGEYIKLKVIGQDSSE-IHFKVKMTTH
Sp_SUMO 1 --MSESPSANISDADKSAITPTTGDTSQQDVKPSTEHINLKVVGQDNNE-VFFKIKKTTE
Sp_SLD1 192 HSKSDHSTLYHSKSEFSTNEPVISVVLQLAVIGQRIPNSNISLPRDWEAPLFFKVKSNQQ


Hs_SUMO-1 44 LKKLKESYCQRQGVPMNSFRFLFEGQRIADNHTPKELGMEEEDVIEVYQEQMGGHSTV--
Sp_SUMO 58 FSKLMKIYCARQGKSMNSLRFLVDGERIRPDQTPAELDMEDGDQIEAVLEQLGGCTHLCL
Sp_SLD1 253 FRRVRIAYSER--KKVDNVVLVFQNQRLWDYGTPKGAGMLKVDTRLVVHAYCHSDFIS--
 ####

SBM2

B

Hs_SUMO-1 1 --------MSDQEAKPSTEDLGD------KKEGEYIKLKVIGQDSSEIHFKVKMTTHLKK
Sp_SUMO 1 MSESPSANISDADKSAITPTTGDTSQQDVKPSTEHINLKVVGQDNNEVFFKIKKTTEFSK
Sp_SLD2 299 AYCHSDFISLKRIKELEVEKLSSVTEDSTAQTCKLITLLLRSSKSEDLRLSIPVDFTVKD


Hs_SUMO-1 47 LKESYCQRQGVPMNS-FRFLFEGQRIADNHTPKELGMEEEDVIEVYQEQMGGHSTV--
Sp_SUMO 61 LMKIYCARQGKSMNS-LRFLVDGERIRPDQTPAELDMEDGDQIEAVLEQLGGCTHLCL
Sp_SLD2 360 LIKRYCTEVKISFHERIRLEFEGEWLDPNDQVQSTELEDEDQVSVVLD----------
 ######
 SBM3

C

~ ~

Sp_SUMO 1 --MSESPSANISDADKSAITPTTGDTSQQDVK-PSTEHINLKVVGQDNNEVFFKIKKTTE
Sp_SLD2 299 --AYCHSDFISLKRIKELEVEKLSSVTEDSTA-QTCKLITLLLRSSKSEDLRLSIPVDFT
Sp_SLD1 192 HSKSDHSTLYHSKSEFSTNEPVISVVLQLAVIGQRIPNSNISLPRDWEAPLFFKVKSNQQ
 ** * ** * *
 ~ ~ $$
Sp_SUMO 58 FSKLMKIYCARQGKSMNS-LRFLVDGERIRPDQTPAELDMEDGDQIEAVLEQLGGCTHLC
Sp_SLD2 357 VKDLIKRYCTEVKISFHERIRLEFEGEWLDPNDQVQSTELEDEDQVSVVLD---------
Sp_SLD1 253 FRRVRIAYSER---KKVDNVVLVFQNQRLWDYGTPKGAGMLKVDTRLVVHAYCHSDFIS-
 * *

Sp_SUMO 117 L
Sp_SLD2 -
Sp_SLD1 -
